# Neighbors Working Together: a *Toxoplasma* Rhoptry Protein That Facilitates Dense Granule Protein Translocation into the Host Cell

**DOI:** 10.1128/mSphere.00523-19

**Published:** 2019-07-31

**Authors:** Gustavo Arrizabalaga

**Affiliations:** a Indiana University School of Medicine, Indianapolis, Indiana, USA

**Keywords:** GRA16, GRA24, MYR1, ROP17, *Toxoplasma gondii*, c-MYC, effectors, kinase, protein translocation

## Abstract

The opportunistic pathogen Toxoplasma gondii is highly adept at manipulating host cell functions. While inside a host cell, *Toxoplasma* divides within a parasitophorous vacuole from which it secretes numerous effector proteins from its dense granules. Many of these so-called GRA proteins are translocated from the parsitophorous vacuole into the host cell where they directly disrupt host signaling pathways. The machinery that drives the translocation of GRA proteins across the parasitophorous vacuole membrane is being elucidated through both genetic and biochemical approaches. A new *mSphere* research article (M. W. Panas, A. Ferrel, A. Naor, E. Tenborg, et al., mSphere 4:e00276-19, 2019, https://doi.org/10.1128/mSphere.00276-19) describes how the kinase ROP17, which is secreted from the parasite’s rhoptries into the host cell during invasion, regulates the translocation of GRA effectors.

## Abstract (precis)

The opportunistic pathogen Toxoplasma gondii is highly adept at manipulating host cell functions. While inside a host cell, *Toxoplasma* divides within a parasitophorous vacuole from which it secretes numerous effector proteins from its dense granules. Many of these so-called GRA proteins are translocated from the parsitophorous vacuole into the host cell where they directly disrupt host signaling pathways.

## COMMENTARY

Toxoplasma gondii is an obligate intracellular parasite with the remarkable ability to infect any nucleated cell in virtually all warm-blooded animals. Not only is *Toxoplasma* highly promiscuous in terms of its host range, it is also highly prevalent due to its multiple routes of transmission: ingestion of environmental cysts expelled in the feces of cats, ingestion of tissue cysts in the meat from infected animals, and congenitally. Part of its success as a parasite and pathogen are due to a large arsenal of effectors that are secreted into the host cell during invasion and intracellular growth (1). These proteins coordinate the manipulation of a vast array of host cell functions including apoptosis, cytoskeleton and organellar arrangement, transcription, and immune response (2).

Secretion of these “master manipulators” is a highly coordinated event involving two unique parasite organelles: rhoptries and dense granules. As it invades, *Toxoplasma* actively invaginates the host plasma membrane to form the parasitophorous vacuole (PV) within which it will divide. During invasion, the rhoptries discharge part of their contents into the host cells. Many of these secreted proteins, known as ROPs, are protein kinases and pseudokinases that co-opt host functions. Once inside the PV, numerous proteins are secreted from spherical organelles known as the dense granules. While some of these GRA proteins stay within the PV space or associate with the PV membrane (PVM), some of them, such as GRA16, GRA18, and GRA24, are translocated across the PVM into the host cytosol where they affect various host cell signaling pathways. How these effectors move from the PV to the host cytosol is a mystery that has recently begun to be unraveled. In particular, three parasite proteins, MYR1, MYR2, and MYR3, have been identified as being part of a putative translocation complex (3, 4). In a new report in *mSphere* (5), Panas et al. describe how the rhoptry serine/threonine kinase ROP17, which is secreted during invasion and associates with the host cytosol side of the PV, is required for translocation of GRA effectors and the ensuing host signaling disruptions. This discovery serves as an example of functional cooperation between proteins secreted from different organelles, and it represents the first enzymatic function identified as critical for GRA protein translocation.

The identification of ROP17 as a regulator of translocation comes as an elegant extension of the forward genetic approaches that identified MYR1, MYR2, and MYR3 (3, 4). Among the host proteins affected by *Toxoplasma* is the oncogenic factor c-MYC, which is actively upregulated at the protein level in infected host cells (6). To begin dissecting the mechanisms behind *Toxoplasma*’s manipulation of host signaling events, Franco et al. devised a forward genetic approach to identify mutant parasites unable to induce c-MYC in human bone marrow macrophages (3). This genetic scheme relied on fluorescence-activated cell sorting (FACS) to select *Toxoplasma*-infected cells in which a GFP−c-MYC reporter failed to be upregulated. Whole-genome sequencing of three of the multiple isolated mutant clones identified the three so-called Myc regulation (MYR) genes as required for c-MYC induction and translocation of GRAs from the PV into the host cell (3, 4). MYR1, -2, and -3 associate with the periphery of the PV with MYR1 and MYR3 forming a complex at the PVM (4). It has been proposed that these three proteins are part of a translocon system that allows movement of GRA proteins across the PVM, but based on translocation systems in other organisms, additional proteins are likely required in this process. With this in mind, the authors went back to their collection of mutant clones with c-MYC upregulation defects to identify novel components of the translocation machinery. Specifically, they interrogated each mutant clone for its ability to translocate epitope-tagged GRA16 and GRA24 into the host cell. Since the *myr1*, *myr2*, and *myr3* mutants show a complete disruption in translocation of GRAs, they focused attention on one mutant with a partial defect on GRA16 and GRA24 translocation with the intention of identifying new genes. This simple and elegant approach paid off: whole-genome sequencing of the mutant revealed a missense mutation in ROP17 (5). Complete knockout of ROP17 confirmed its role in GRA protein translocation and in the host cell gene expression changes imparted by GRA effectors. Interestingly, the authors showed that ROP17 kinase activity is required for these functions, which suggest that this secreted kinase might function through phosphorylation of components of the translocon machinery.

ROP17 was initially identified as a member of a complex of secreted rhoptry kinases and pseudokinases (7). In conjunction with ROP18, ROP17 is involved in the inactivation of murine immunity-related GTPases (IRGs), which, if active, can attack the PVM and neutralize the parasites (7). Thus, ROP17 appears to play at least two distinct roles: inactivation of IRGs and control of GRA translocation. This new role for ROP17 calls for a reevaluation of our previous understanding of published results. For instance, it had been reported that knockout of ROP17 attenuates virulence in mice (7). The attenuated virulence may be due to a lack of IRG inactivation, but a defect in translocation of GRA effectors is also likely to contribute to this phenotype.

How ROP17 influences translocation of GRA proteins is still a pending question for which an answer will require further investigation and, likely, a more comprehensive understanding of all the components of the translocon complex. Since simply mutating the catalytic residues of ROP17 disrupts translocation, it is reasonable to propose a model by which ROP17 phosphorylates components of the translocon that are required for its function. Furthermore, Panas et al. (5) show that the action of ROP17 on GRA protein translocation takes place on the host cytosolic side of the PVM. As the components and the structure of this novel translocon are deciphered, the substrates of ROP17 and other regulatory proteins will become clear, shedding light on how the many arrows with which *Toxoplasma* hits its target move from the PV into the host cytosol.
